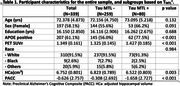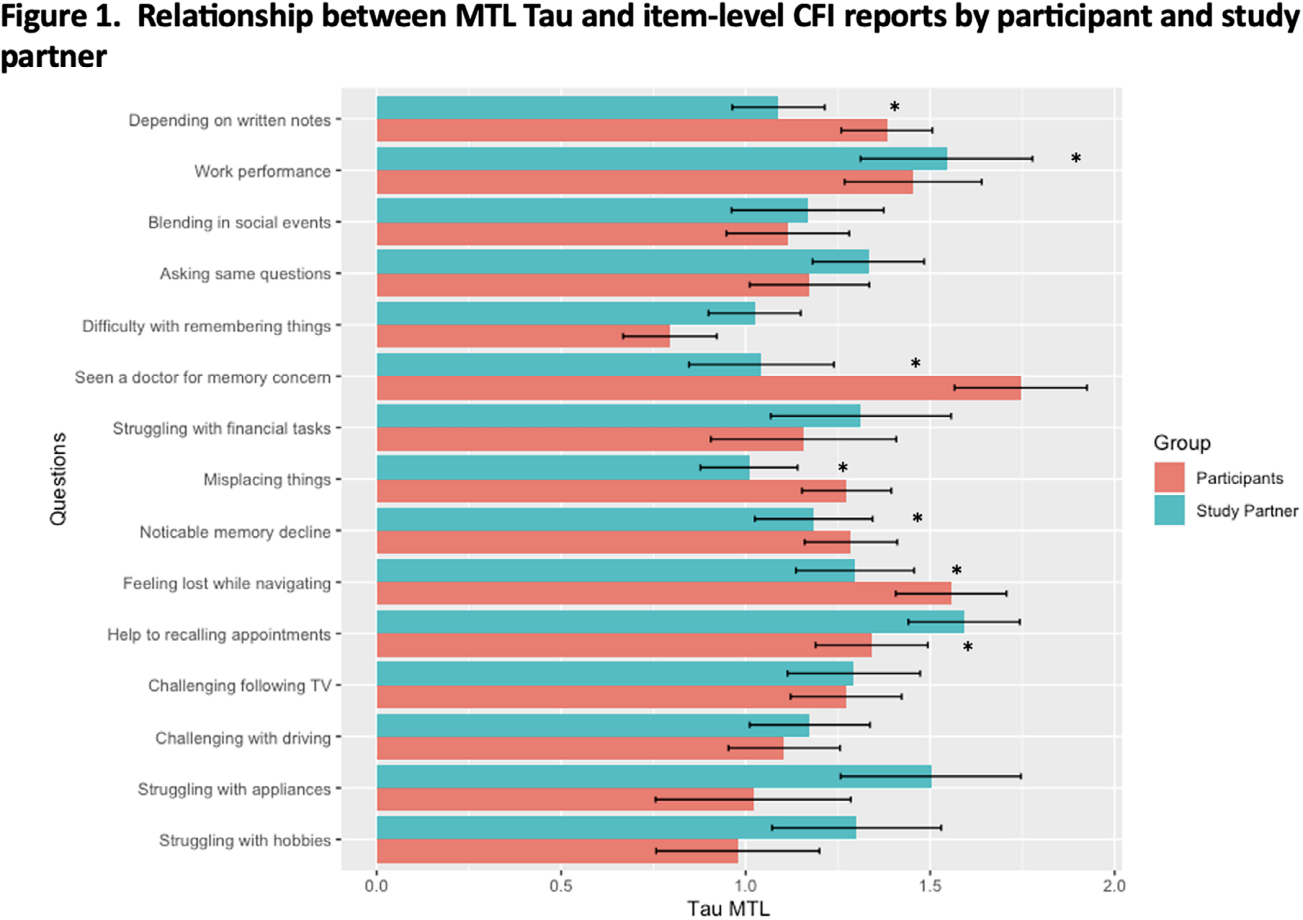# Linking Cognitive Function Index Item‐Level Responses to Tau Pathology in Amyloid Positive Cognitively Unimpaired individuals: Findings from The A4 Study

**DOI:** 10.1002/alz.093283

**Published:** 2025-01-03

**Authors:** Idris Demirsoy, Kellen K. Petersen, Bhargav Teja Nallapu, Elham Ghanbarian, Richard B. Lipton, Laura Rabin, Ali Ezzati

**Affiliations:** ^1^ University of California, Irvine, Irvine, CA USA; ^2^ Usak University, Usak Turkey; ^3^ Albert Einstein College of Medicine, Bronx, NY USA; ^4^ Brooklyn College of the City University of New York, Brooklyn, NY USA

## Abstract

**Background:**

The Cognitive Function Index (CFI) is a validated test used to assess changes in self‐perceived cognitive and functional status as reported by an individual and their study partner. Previous studies have demonstrated an inverse correlation between higher amyloid‐beta (Aβ) burden and CFI, with certain CFI items exhibiting stronger associations than others. However, there is limited understanding of the association between declines in cognition and function, as assessed by CFI, and Tau levels measured by PET.

**Methods:**

Participants were 339 cognitively unimpaired, Aβ positive, individuals enrolled in the Anti‐Amyloid Asymptomatic Alzheimer’s (A4) Study who underwent tau‐PET imaging. Participants were classified as tau‐PET positive (T+) or negative (T‐) based on tau levels in the medial temporal lobe (T_MTL_^+^/T_MTL_^‐^). Participants and their study partners assessed subjective changes in cognition and function over the past year using a 15‐item CFI questionnaire. For each CFI item, the relationship between Tau and CFI reports (Yes/Maybe vs No) was investigated using logistic regression models.

**Results:**

Participants were on average 72.38(SD = 4.87) years old, 58.1% were female, and 23.6% was T_MTL_^+^. Table 1 summarizes the sample characteristics. Higher T_MTL_ was significantly associated with participant report of decline on six items, seeing a doctor about memory concerns (OR = 1.746, p = 0.002) and getting lost while travelling to another city (OR = 1.557, p = 0.003) showing the highest odds ratios. By contrast, Higher T_MTL_ was associated with study partner report of decline on only one item: needing help from others to remember appointments/occasions (OR = 1.592, p = 0.002).

**Conclusion:**

Unique CFI items were associated differentially with higher T_MTL_ for participants and their study partners. The value of the CFI questionnaire with participant as the source of information may be higher than responses provided by informant in terms of the association with Tau‐PET positivity. This is consistent with the idea that self‐awareness of cognitive decline might precede the observations made by others.